# The impact of a family web-based nutrition intervention to increase fruit, vegetable, and dairy intakes: a single-blinded randomized family clustered intervention

**DOI:** 10.1186/s12937-022-00825-6

**Published:** 2022-12-20

**Authors:** Vicky Drapeau, Andrée-Anne Harvey, Raphaëlle Jacob, Véronique Provencher, Shirin Panahi

**Affiliations:** 1 Department of Physical Education, Laval University, Quebec, Quebec, Canada; 2 Centre Nutrition, santé et société (NUTRISS), Institute of Nutrition and Functional Foods (INAF), Laval University, Quebec, Quebec, Canada; 3 Quebec Heart and Lung Institute Research Center, Quebec, Quebec, Canada; 4 Centre recherche interuniversitaire sur la formation et la profession enseignante (CRIFPE-Laval), Laval University, Quebec, Canada; 5 School of Nutrition, Laval University, Quebec, Quebec, Canada

**Keywords:** Family-based intervention, Childhood obesity, Fruits and vegetables, Dairy products, Healthy eating, Diet quality

## Abstract

**Background:**

The importance of adopting healthy eating habits at a young age to prevent obesity and chronic diseases justifies the need for effective interventions.

**Objective:**

This study evaluated the impact of a family web-based nutrition intervention on vegetable and fruit (V/F) and dairy product (DP) consumption, nutrient intakes, diet quality and BMI or BMI z-scores.

**Methods:**

Forty-three families with children aged 8–16 years were randomized to either the family web-based intervention, or web-based general nutrition guidelines (control) over 8 weeks. Nutritional variables were assessed with three-day dietary records while anthropometry (body weight and height) was assessed with standardized measures at baseline (PRE), immediately after the intervention (POST 1) and 3–6 months after the intervention (POST 2). Linear mixed models for repeated measures were used to assess the main effects and their interactions followed by post hoc tests.

**Results:**

The intervention had an effect on DP, total sugar, potassium, magnesium, and calcium in children (Group x Time, *P* = 0.02 to 0.03) and on DP, V/F juice, carbohydrates, total sugar, saturated fat, protein and calcium in parents (Group x Time, *P* = 0.01 to 0.03). Post hoc tests revealed children in the intervention group increased their DP intakes immediately after the intervention (POST1) but decreased at follow-up (POST2). No effect of the intervention on V/F, diet quality or BMI was observed.

**Conclusion:**

Compared to general nutrition guidelines, this family web-based nutrition intervention had a modest effect on nutrient intakes, but beneficial effect on DP intakes in the short term.

**Trial registration:**

ClinicalTrials.gov, NCT03798808, Registered 10 january 2019 - Retrospectively registered.

**Supplementary Information:**

The online version contains supplementary material available at 10.1186/s12937-022-00825-6.

## Introduction

Promoting healthy eating habits at a young age represents one key solution for the prevention and treatment of childhood obesity and other chronic diseases [1]. Factors such as the family environment (e.g. parental influence, role modeling, and food availability at home) are important determinants of children’s eating habits and obesity [2–4]. Many parents and family-focused childhood obesity prevention and management programs have been developed over the last few decades. A systematic review and meta-analysis highlighted a modest, but significant effect of these interventions on body mass index (BMI) or BMI z-scores [4]. The focus of efficient behavioural interventions varied between studies and included nutrition, physical activity education sessions, behavioural therapy or a combination of these [4]. Greater parental involvement, longer duration [4] and inclusion of different behavioural change techniques such as prompt barrier identification, goal settings, self-monitoring and changes in the home environment, [2] and positive parenting skills such as role modeling and encouraging healthy eating/exercise behaviours in children or the whole family [5] were associated with intervention effectiveness. Simple dietary messages specifically targeting parents and regular follow-ups were also found to be beneficial in improving vegetable and fruit (V/F) consumption and reducing fat intakes in children [6]. Thus, parental involvement, behavioural change techniques, and parent-targeted strategies seem to be important intervention strategies in childhood obesity prevention and management programs. However, as indicated in a recent literature review and meta-analyses, more research is needed on childhood obesity prevention and management programs due to a lack of high-quality evidence [5].

More recently, the use of web or technology-based interventions (also called eHealth interventions, i.e. using the internet, telephone, social media, emails) has shown promising results [7, 8]. These modalities of intervention are generally more attractive and interactive, usually offer individual feedback and strategies, are flexible and cost-effective, which ultimately may result in higher engagement of participants in the context of childhood obesity prevention and management [9]. The systematic review and meta-analysis from Hammersley et al. (2016), on the effectiveness of parent-focused childhood and adolescent eHealth interventions, demonstrated that while most studies have shown no effect on BMI or BMI z-scores, some studies found improvements in at least one dietary or physical activity outcome compared to control [10]. Although, these studies have examined interventions combining various modalities such as web or technology-based interventions and face-to-face or group sessions, most of the interventions targeted primarily parents and their quality was generally not high [10]. The most efficient strategies used in eHealth interventions have not been specifically addressed in this review. In another recent systematic review and meta-analysis primarily aimed at promoting positive parental feeding practices with web-based interventions, the most common behavioural changed techniques used were instruction and demonstration on how to perform the behaviour (role model) while increased food availability and accessibility was the only parental practice for which the effect was significant [11]. These reviews also highlight the heterogeneity and low quality of the studies which make it difficult to identify the most efficient key features and specific targets [10, 11]. Thus, evidence shows that families and web-based interventions designed to promote healthy eating patterns in children are a promising approach to increase parents’ engagement and facilitate behaviour change in children. Although specific key features are not yet identified, based on face-to-face family interventions presented above, these web-based interventions should favor parental engagement and include behavioural change techniques such as self-monitoring, feedback, reminders, and social support as much as possible.

Among the healthy eating habits that could be targeted in family web-based interventions to prevent or treat childhood obesity, V/F and dairy product (DP) consumption represent a positive approach to improve diet quality and decrease energy density. Several studies have demonstrated the beneficial effects of V/F on overall diet quality and body weight control in adults [12, 13]. Whole fruit and low-fat milk consumption were also associated with better body weight control in adults from the Québec Family Study [14]. Although the relationship between V/F and adiposity is unclear in children [15], lowering energy density by incorporating V/F into children’s meals may help to decrease overall energy intake [16]. Increasing V/F or DP in children has also been shown to decrease the consumption of unhealthy snacks/foods in school-based interventions [17–19] and high-fat/high-sugar foods in family-based interventions [20]. Furthermore, DP intake, particularly milk consumption, has been associated with a lower risk of obesity [21, 22].

This study aimed to assess the effectiveness of a family web-based nutrition program, called Family Nutriathlon, on V/F and DP consumption as primary outcomes, and on nutrient intakes, diet quality, and anthropometric measures as secondary outcomes. We hypothesized that compared to general nutrition guidelines, children and their parents in the intervention group will improve V/F and DP consumption and diet quality and that children living with overweight or obesity will have a lower increase in BMI z-score over time.

## Methods

### Study design

In an eight-week, single-blinded, randomized, family-clustered, controlled intervention, eligible families were randomized to either the Family Nutriathlon intervention or control (general nutrition guidelines based on the 2007 Canada’s Food Guide). All families participated in three testing sessions, at baseline (PRE), immediately after the intervention week 9; (POST 1) and 3–6 months after the intervention (POST 2) where outcome measures were assessed. The nutrition guidelines (intervention or control) were explained first in a face-to-face 60-minute period with the dietitian followed by all regulation periods/sessions that were conducted online.

### Participants

Families were recruited between February 2016 and May 2017 via emails sent to the Université Laval community list and then with a flyer to primary schools. After the first year of recruitment, the advertisement was sent to nutrition clinics and family medicine groups in the Quebec City area. Family inclusion criteria were: having children aged 8–16 years, at least one child with BMI percentile ≥85th for age according to the growth charts from the World Health Organization) [23], parents with stable body weights (±2 kg) over the previous 2 months, parents with full custody of children and Internet access at home. Exclusion criteria were: parent or child using medications influencing appetite or weight control (including medications for attention deficit disorders), suffering from any chronic diseases (except hypertension and dyslipidemia), having food allergies or restrictions concerning V/F and DP and following a specific diet (e.g. vegetarianism). Although having a child with overweight or obesity was an inclusion criterion, this information was not included in any advertisement during recruitment nor mentioned during the intervention to minimize the risk of stigmatization of the child and promote an intervention that targets the entire family. Eligibility was first evaluated by telephone with one parent. If families matched the criteria, an in-person meeting with the family was planned to confirm eligibility (e.g. body weight, height, BMI z-score).

Among all the families recruited to take part in this study, 13 families came from a previous Family Nutriathlon pilot study. These families (*n* = 13; intervention group *n* = 9 and control group *n* = 4) were recruited between November 2012 and September 2013. This pilot study was conducted to validate the feasibility of Family Nutriathlon while providing preliminary data. The methodology between the two studies was identical with the exception that families were not recruited based on children’s BMI z-scores and the follow-up period was 3 months. According to Charlesworth et al. (2013) and Thabane et al. (2010), the pilot study can be pooled with the main study when there are only minor changes between a pilot and main study in order to increase efficiency of a main study, reducing the cost, time, burden on the study population, and increasing the sample size [24, 25].

The study (main and pilot) was approved by the Research Ethics Board at Université Laval. All parents and children gave their written informed consent and assent, respectively, to participate in this study. Families received financial compensation for their participation. After eligibility assessment and consent, families were randomized to the intervention or control groups. The randomization was performed using the random function of Excel, with two blocks of 20 families. Since the control group received minimal intervention, families were not aware of which group they were assigned to.

### Intervention

Family Nutriathlon was an eight-week, family-based nutrition program where parents and their children were encouraged to increase the quantity and variety of V/F and DP consumption. This program was based on another school-based nutrition program called Team Nutriathlon developed by our research group, which was found to positively impact V/F and DP intakes in elementary [26] and secondary school students [27]. Based on our past experience with Team Nutriathlon, we used the same duration for the Family Nutriathlon in order to include three formal regulation periods/sessions with a registered dietitian to modify their behaviors. The implementation of the pilot project on Family Nutriathlon confirmed that this duration was convenient for most families considering their time constraints. Family Nutriathlon includes behaviour change techniques related to the self-determination theory such as goal-setting, self-monitoring, feedback, identification of barriers, solutions (competence) and social support (relatedness) [28]. It was also designed to develop the autonomy of parents and children (i.e. decision-making and control over their food choices) towards the gradual adoption and maintenance of healthy eating habits. Additional details regarding the web-based platform have been published elsewhere [27].

More specifically, Family Nutriathlon included individual and team goals for the quantity and variety of V/F and DP. The individual goals required participants to attain a specific quantity of servings per day (i.e. 6 servings of V/F and 3 servings of DP), based on the 2007 Canadian Food Guide [29]. The team goals were based on both quantity and variety. For the “quantity” goal, each family must have reached a certain amount of servings of V/F and DP, depending on the number of family members (i.e. larger families had higher goals). For the “variety” goal, the consumption of V/F and DP had to be equal among the six categories of colors according to their nutrient content. The “variety” goal aimed to increase the likelihood that participants would taste new V/F and DP during the program. During the program, participants were asked to report their daily consumption of V/F (including 100% pure juices) and DP for each meal and weekday on a web-based platform. This self-monitoring process enabled the production of digital summary reports every two weeks including information on the number and variety of servings consumed. These reports were analyzed during three regulation periods (or sessions) where families met with a registered dietitian via an online program (e.g. Skype) to have a formative evaluation of their behaviors over the previous two weeks and reflect on the level of attainment of the Family Nutriathlon goals. During these sessions, all family members were encouraged to identify barriers to V/F and DP intakes and strategies to maintain or increase their V/F and DP consumption to fulfill the goals of Family Nutriathlon with the help of a dietitian. Then, families were invited to plan and take action to modify their eating habits. Digital booklets created by dietitians and food designers containing recipes and strategies on ways to cook more meals with V/F and DP, eating family meals, and purchasing less expensive V/F and DP were provided to families.

Participants in the control group received an online standard nutrition intervention (i.e. recommendations based on the 2007 Canada’s Food Guide) [29]. They were not informed that they were following the Canadian guidelines. Instead of being encouraged to specifically increase their consumption of V/F and DP, they received general advice to eat more fresh V/F every day, choose less processed cereals, choose good protein sources such as low-fat milk, yogurt, cheese or a soy beverage every day and choose good sources of fat. They were not exposed to the Family Nutriathlon objectives or the website but they received the same number of online sessions (*n* = 3) with a registered dietitian as the intervention group.

### Measurements

Primary outcomes: Servings of V/F and DP and nutrient intakes were measured using three-day dietary records [30]. The mother generally completed them for the children at home. Dietary records were entered into the Nutrific software (Laval University, QC, Canada), which was linked to the Canadian Nutrient File database [31] to analyze nutrient intakes. Servings of V/F and DP were derived from these records using those established by the 2007 Canadian Food Guide (e.g. 1 apple = 1 serving of fruit; 1 cup of milk = 1 serving of DP) [29]. At baseline, parents also completed a socio-demographic questionnaire. Diet quality was assessed using the Nutrient-Rich Foods (NRF9.3) index, calculated based on 9 nutrients to encourage (protein, fiber, vitamins A, C and E, calcium, iron, potassium, and magnesium) and 3 nutrients to limit (sugar, saturated fat and sodium) and provides a validated tool to assess the quality of the diet, foods or meals [32, 33]. Each family completed a one-day dietary record before each regulation period/session as a compliance strategy. Compliance was assessed by family attendance to sessions with the dietitian.

Secondary outcomes: Because this intervention was relatively short, the impact of the intervention on BMI and BMI z-scores was considered secondary. Body weight (kilograms) and height (meters) were objectively measured using a bioimpedance balance (TANITA, model TBF-310) and a stadiometer according to the Airlie procedures [34]. Body mass index (BMI) was calculated as the weight (in kilograms) divided by height squared (in meters). Children’s BMI z-scores were calculated according to age and sex growth charts from the World Health Organization [23]. Children were identified as healthy weight (5th–85th percentile) or overweight and living with obesity (≥85th BMI percentile for age and ≥ 97th BMI percentile for age, respectively).

### Statistical analysis

Statistical analyses were performed using JMP analysis software (JMP 14.0, SAS Institute, Cary, NC, USA), and SAS studio v. 9.04 (SAS Institute, Cary, NC, USA). The sample size was calculated based on a mean increase of 0.5 serving/day in V/F consumption in parents and children estimated from our pilot study and the mean observed in previous studies [35–37]. Thus, to detect a statistical difference between groups (alpha level of 0.05 and power of 0.80), a total of 34 families (i.e. mean of 2 parents and 2 children) were required. Based on an estimated attrition rate of 15%, we aimed to recruit 40 families (20 in each group). Group differences in baseline participant characteristics were assessed using Chi-Square or Student t-tests. Linear mixed models for repeated measures were performed to assess the main effect of group, time, and their interactions, using time, group and their interaction as fixed effects and participants and families as random effects. A compound symmetry, autoregressive, or unstructured covariance matrix was used for each model depending on the Akaike Information Criterion. Changes in BMI z-scores between groups were assessed in all children and children with BMI percentiles ≥85th for age. If an interaction was observed, Tukey-Kramer’s post hoc test was performed to identify differences. Analyses in parents were adjusted for baseline energy intake, although the difference in baseline energy intake (300 kcal) was not statistically different between groups (*P* = 0.09). Because of differences in baseline characteristics between families recruited from the pilot vs the main study (data not shown), all analyses have been adjusted for the study project (pilot vs main study) using an indicator variable (main study, 0; pilot study, 1). The data are expressed by means ± standard deviation (SD) and adjusted means (95% confidence interval) unless otherwise indicated, and a *P*-value < 0.05 was considered significant.

## Results

### Participant characteristics

**Fig. 1:**
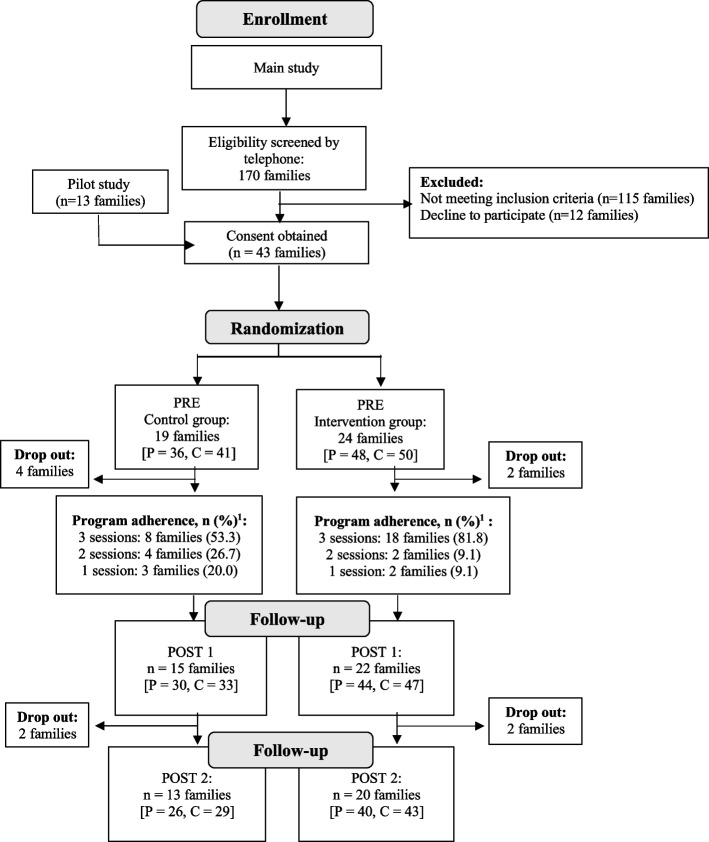
Flow diagram. P: Parents, C: Children, PRE: Before the intervention, POST 1: immediately after the intervention, POST 2: 3 to 6 months after the intervention. ^1^Adherence: number of regulation periods/sessions attended by each of the families on a total of three sessions

A total of 43 families participated in this study (control group *n* = 19; intervention group *n* = 24) (Fig. 1). The mean number of participants per family was 4 members for both groups. There were no significant differences between groups at baseline for anthropometric measures (BMI and BMI z-scores) in parents or children (Table 1).

**Table 1:** Baseline Characteristics of Parents and their Children

	Parents			Children		
	Control	Intervention	*P*	Control	Intervention	*P*
*n*	31	44		39	48	
Age, y	40.3 ± 4.3	41.8 ± 4.2	0.12	10.7 ± 2.2	11.2 ± 2.7	0.30
Sex, % female (n)	54.8 (17)	50.0 (22)	0.68	53.8 (21)	37.5 (18)	0.13
BMI	28.6 ± 4.0	28.2 ± 4.5	0.71	20.6 ± 3.3	20.5 ± 3.5	0.84
BMI z-score	–	–	–	1.1 ± 0.8	1.0 ± 0.8	0.31
Overweight, % (n)	32.3 (10)	43.2 (19)	0.48	41.0 (16)	43.8 (21)	0.57
Obesity, % (n)	45.2 (14)	31.8 (14)		12.8 (5)	6.3 (3)	
V/F intake, servings	5.8 ± 2.4	6.3 ± 3.4	0.48	5.2 ± 2.9	5.3 ± 2.5	0.81
DP intake, servings	1.8 ± 1.0	1.6 ± 0.8	0.42	2.0 ± 1.0	2.0 ± 1.1	0.10
Reaching V/F recommendations, % (n)^1^	34.6 (9)	43.2 (19)	0.48	34.3 (11)	37.5 (18)	0.78
Reaching DP recommendations, % (n)^2^	11.5 (3)	2.3 (1)	0.11	25.0 (8)	16.7 (8)	0.36
**Family income** (CAN$), % (n)						
< $49,999	3.2 (1)	7.0 (3)	0.47			
$50,000-99,000	35.5 (11)	18.6 (8)				
> $100,000	61.3 (19)	74.4 (32)				
**Education level**, % (n)						
High School or Professional Diploma	22.5 (7)	9.1 (4)	0.57			
Diploma of College Studies	22.3 (7)	18.2 (8)				
University Diploma	51.6 (16)	68.2 (30)				
Others	3.2 (1)	4.6 (2)				
Number of participants per family	4.5 ± 0.6	4.3 ± 0.6	0.05			

Values are presented as means ± standard deviation unless indicated otherwise

V/F, vegetables and fruit including fruit juice; DP, dairy products

^1^V/F recommendations based on the 2007 Canada’s Good guide are as follows: Children aged 4–8 y: 5 servings/day; Children aged 9–13 y: 6 servings/day; Girls and boys aged 14–18 y: 7 and 8 servings/day, respectively; Women and men aged 19–50 y: 7–8 and 8–10 servings/day, respectively

^2^DP recommendations based on the 2007 Canada’s Food Guide are as follows: Children aged 4–8 y: 2 servings/day; Children aged 9–18 y: 3–4 servings/day; Adults aged 19–50 y: 2 servings/day

### Children’s V/F and DP consumption, nutrient intakes, diet quality and BMI z-scores

**Fig. 2:**
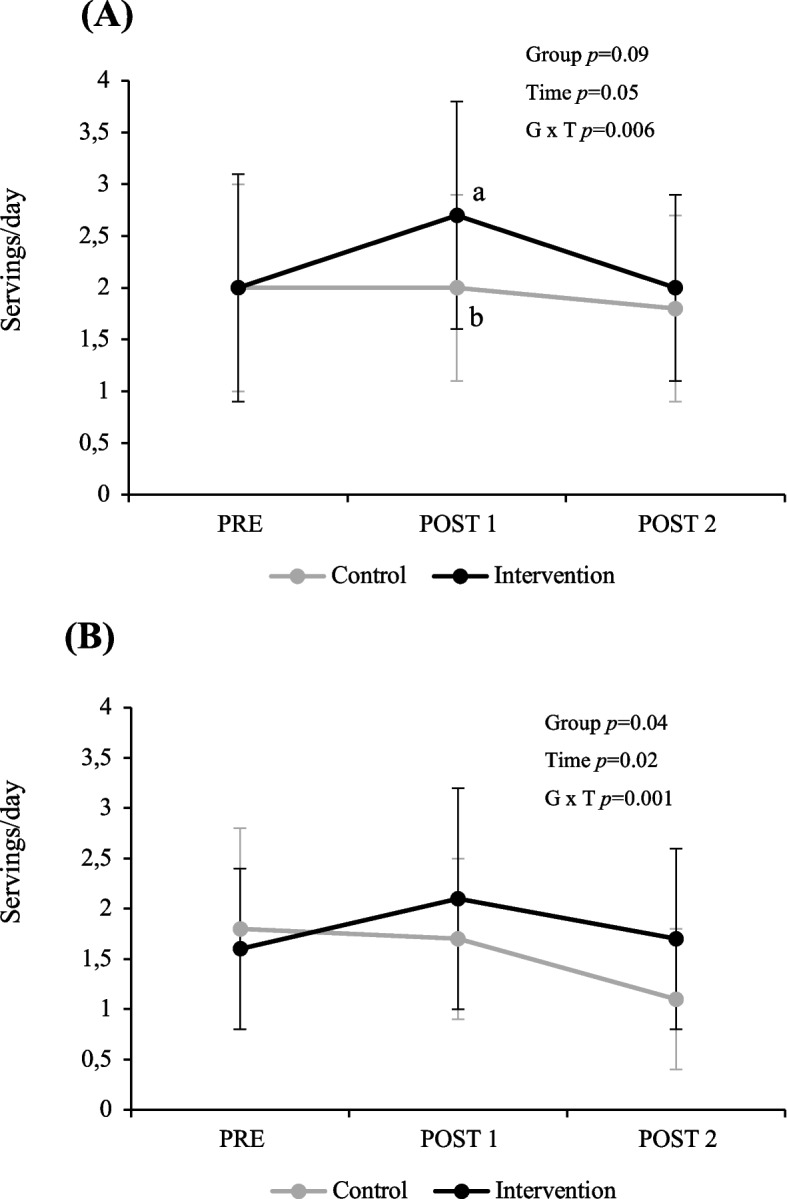
Daily DP intakes for (**A)** children and (**B)** parents at baseline (PRE), immediately after (POST 1) and 3–6 months after the intervention (POST 2). Values are presented as means ± standard deviations; Linear mixed models for repeated measures were performed to assess the main effect of group, time and their interactions. Analyses have been adjusted for study project (pilot vs main study). Participants as well as families were added as a random effect. Parents' analyses were adjusted for energy intake baseline values. When an interaction was observed, the Tukey-Kramer’s post hoc test was performed. Different letters (a, b) indicate between-group differences at each time point (*P* < 0.05). Difference vs. control PRE: − 0.1 (− 0.8, 0.7), POST 1: 0.8 (0.01, 1.6) and POST 2: 0.4 (− 0.4, 1.1) for children and PRE: − 0.1 (− 0.8, 0.6), POST 1: 0.6 (− 0.2, 1.4) and POST 2: 0.7 (− 0.04, 1.5) for parents

Mean baseline V/F consumption in children was 5.2 ± 2.9 and 5.3 ± 2.5 servings/day in the control and intervention groups, respectively, while DP consumption was 2.0 ± 1.0 and 2.0 ± 1.1 servings/day in the control and intervention groups, respectively. In children, there was no significant effect of the intervention on V/F consumption, yet, the intervention group significantly increased their DP intakes compared to the control group (*P* group x time = 0.006, Fig. 2). Post hoc analyses revealed that children in the intervention group significantly increased their DP intakes compared to the control group in POST 1 (Tukey-Kramer, *P* < 0.05). This increase in DP consumption was not maintained from POST 1 to POST 2 in children in the intervention group, such that DP consumption at POST2 was no longer significantly higher than baseline values (POST 1 vs. PRE, estimate 0.73, 95% CI 0.25 to 1.22, *P* = 0.0004; POST 2 vs. PRE, estimate 0.20, 95% CI − 0.29 to 0.70). There was a significant group x time interaction for total sugar, potassium, magnesium, and calcium (*P* = 0.02 to 0.03), but no effect on overall diet quality (Table 2). In children, there was a significant decrease in BMI z-scores over time (*P* = 0.03) in both groups, but no group x time interaction (Table 4). Similar results were observed with children who were overweight and with obesity only (data not shown).

**Table 2:** Daily Vegetables and Fruit Mean Intakes, Nutrient Intakes and Diet Quality for Children

	Control^1^			Intervention^1^			Difference vs. control^2^			*P* value		
	PRE	POST 1	POST 2	PRE	POST 1	POST 2	PRE	POST 1	POST 2	Group	Time	G x T
*n*	32	24	28	48	38	36						
***V/F intakes***												
V/F, servings	4.1 ± 2.5	4.3 ± 2.2	4.6 ± 2.3	3.9 ± 2.1	4.9 ± 2.2	4.5 ± 1.9	0.1 (− 1.8, 1.9)	1.1 (− 0.8, 3.1)	0.6 (− 1.3, 2.6)	0.32	0.09	0.10
Juice, servings	1.1 ± 1.5	1.2 ± 1.4	1.0 ± 1.2	1.4 ± 1.4	1.5 ± 1.2	1.5 ± 1.4	0.1 (− 1.0, 1.2)	0.03 (− 1.0, 1.1)	0.4 (−0.6, 1.4)	0.59	0.82	0.50
***Nutrient intakes and diet quality***												
Energy, kcal	2159.6 ± 554.5	1806.0 ± 371.9	1892.7 ± 342.9	2124.6 ± 555.2	1920.4 ± 425.0	1982.0 ± 430.0	−14.5 (− 431.9, 403.0)	198.0 (− 190.5, 586.4)	124.9 (− 256.1, 506.0)	0.39	< 0.0001	0.19
Carbohydrate, g	271.5 ± 90.7	225.5 ± 56.7	239.6 ± 49.0	272.1 ± 66.3	252.7 ± 60.4	253.2 ± 57.0	3.7 (− 55.3, 62.8)	−38.3 (− 14.1, 90.8)	23.2 (− 27.4, 73.8)	0.19	0.0004	0.06
Carbohydrate, %	49.7 ± 8.1	49.6 ± 4.7	50.8 ± 6.9	51.7 ± 5.4	52.7 ± 5.1	51.2 ± 4.6	1.8 (− 2.9, 6.6)	2.4 (− 2.7, 7.5)	1.0 (−4.1, 6.1)	0.24	0.89	0.69
Total sugars, g	111.8 ± 49.3	93.0 ± 30.4	101.3 ± 27.6	105.5 ± 30.0	109.7 ± 33.5	106.7 ± 30.0	− 6.4 (− 37.4, 24.6)	20.0 (−9.1, 49.0)	9.7 (−17.3, 36.6)	0.37	0.24	**0.02**
Fat, g	80.6 ± 22.5	65.3 ± 15.1	67.7 ± 17.5	77.8 ± 29.8	62.4 ± 17.4	69.1 ± 19.8	− 2.0 (− 21.2, 17.3)	0.7 (− 17.0, 18.4)	1.6 (− 16.7, 20.0)	0.98	< 0.0001	0.88
Fat, %	33.9 ± 5.9	32.5 ± 3.4	32.1 ± 5.0	32.5 ± 5.3	29.2 ± 4.5	31.2 ± 4.5	− 1.3 (− 5.3, 2.6)	−2.6 (− 6.9, 1.7)	−1.2 (− 5.4, 3.0)	0.16	0.004	0.55
Saturated Fat, g	27.4 ± 9.3	24.3 ± 7.4	23.4 ± 8.5	28.1 ± 10.1	24.2 ± 9.0	26.5 ± 9.1	0.8 (−7.2, 8.7)	1.5 (−7.9, 10.9)	3.3 (− 5.6, 12.2)	0.48	0.04	0.60
Protein, g	86.9 ± 23.1	78.9 ± 15.5	81.0 ± 22.3	83.9 ± 23.2	87.0 ± 23.9	86.8 ± 22.7	−1.9 (− 21.0, 17.1)	11.3 (− 9.0, 31.7)	5.5 (− 14.7, 25.6)	0.42	0.86	0.05
Protein, %	16.4 ± 3.7	17.9 ± 3.6	17.1 ± 3.2	15.9 ± 2.3	18.1 ± 2.7	17.6 ± 3.0	− 0.5 (− 3.0, 2.1)	0.2 (−2.6, 3.0)	0.1 (− 2.9, 3.1)	0.94	< 0.0001	0.70
Fiber, g	20.6 ± 8.0	17.1 ± 5.4	20.7 ± 8.7	19.0 ± 5.9	18.3 ± 5.6	19.1 ± 5.0	− 0.7 (− 6.0, 4.6)	3.0 (− 2.6, 8.7)	0.1 (− 5.5, 5.6)	0.63	0.003	0.06
Potassium, mg	2788.5 ± 841.3	2670.2 ± 714.1	2759.7 ± 591.7	2740.2 ± 619.5	2930.9 ± 812.5	2826.9 ± 620.0	− 27.4 (− 618.9, 564.1)	427.0 (− 205.6, 1059.6)	215.2 (− 406.9, 837.3)	0.28	0.99	**0.03**
Magnesium, mg	311 ± 106.7	268 ± 72.6 ^†^	288.2 ± 80.7	288.7 ± 72.7	275.2 ± 73.7	276.2 ± 63.9	− 15.4 (− 82.0, 51.3)	33.7 (− 36.6, 104.1)	3.4 (− 66.0, 72.8)	0.74	0.001	**0.02**
Calcium, mg	1051.1 ± 363.8	934.1 ± 277.8	986.0 ± 311.1	1054.3 ± 374.1	1179.6 ± 355.2	1031.5 ± 310.8	−6.8 (− 257.5, 243.9)	272.1 (− 5.8, 550.0)	78.2 (− 192.5, 348.9)	0.13	0.73	**0.02**
Vitamin C, mg	127.1 ± 80.2	147 ± 70.8	144.3 ± 62.6	139.6 ± 83.5	155.7 ± 73.9	150.9 ± 72.3	8.6 (− 47.5, 64.8)	5.0 (− 56.6, 66.7)	6.6 (− 54.3, 67.5)	0.68	0.30	0.98
NRF 9.3	43.1 ± 8.7	46.2 ± 5.4	47.6 ± 8.1	42.1 ± 6.5	44.6 ± 5.5	43.6 ± 7.9	− 0.6 (− 6.5, 5.3)	−0.5 (− 6.9, 5.9)	2.8 (− 9.0, 3.4)	0.50	0.001	0.40

*PRE* before the intervention (baseline), *POST 1* immediately after the intervention (week 9), *POST 2* 3–6 months after the intervention, *G x T* Group by time interaction, *NRF* Nutrient Rich Food Index, *V/F* whole vegetables and fruits, *DP* Dairy products; *POST 1* four families did not complete their 3-day dietary record. *POST 2* three families did not complete their 3-day dietary record

^1^Values are presented as means ± standard deviation

^2^Values are presented as adjusted mean (95% confidence interval)

^†^Within-group significant differences vs. PRE

### Parent’s V/F and DP consumption, nutrient intakes, diet quality and BMI

Mean baseline V/F consumption in adults was 5.8 ± 2.4 and 6.3 ± 3.4 servings/day in control and intervention groups, respectively, while DP consumption was 1.8 ± 1.0 and 1.6 ± 0.8 servings/day in control and intervention groups, respectively. Like children, there was no significant effect of the intervention on V/F consumption in parents, but a significant group x time interaction was observed for DP (group x time *P* = 0.001, Fig. 2). Post hoc analyses revealed that parents in the intervention showed an increase in DP at POST1 (POST1 vs PRE, estimate 0.60, 95% CI: 0.05 to 1.12, *P* = 0.03). Group x time interactions were also observed for V/F juice, carbohydrates (g), total sugar, saturated fat, protein (g) and calcium (0.01 ≤ *P* < 0.03) but not for diet quality (Table 3). Post hoc analysis showed no between-group difference in these variables at each time point except for fruit juice which was higher at POST 1 compared to PRE in parents. In parents, there was no significant effect of time, group, or group x time interaction on BMI (Table 4).

**Table 3:** Daily Vegetables and Fruit intakes, Nutrient Intakes and Diet Quality for Parents

	Control^1^			Intervention^1^			Difference vs. control^2^			*P* value		
	PRE	POST 1	POST2	PRE	POST1	POST2	PRE	POST 1	POST2	Group	Time	G x T
*n*	26	20	23	44	34	30						
***V/F and DP intakes***												
V/F*,* servings	5.1 ± 2.2	5.2 ± 2.6	5.0 ± 2.2	5.9 ± 3.3	6.4 ± 2.6	6.3 ± 3.5	0.7 (− 1.7, 3.0)	1.3 (− 1.2, 3.7)	1.0 (− 1.6, 3.6)	0.19	0.87	0.66
Juice, servings	0.7 ± 0.9	0.7 ± 1.1	0.5 ± 0.8	0.4 ± 0.9	1.1 ± 1.2^†^	0.7 ± 1.3	− 0.3 (− 0.9, 0.4)	0.3 (− 0.6, 1.3)	0.2 (− 0.6, 1.1)	0.69	0.16	**0.02**
***Nutrient intakes and diet quality***												
Energy, kcal	2424.1 ± 754.7	2012.0 ± 778.8^†^	2088.1 ± 603.9^†^	2097.5 ± 503.9	2049.5 ± 631.8	2083.4 ± 578.8	−91.9 (− 384.4, 200.6)	282.2 (− 50.0, 614.4)	194.8 (− 130.2, 519.9)	0.09	0.0005	**0.02**
Carbohydrate, g	268.7 ± 81.9	226.3 ± 84.2^†^	235.2 ± 54.3^†^	245.4 ± 66.7	246.6 ± 81.2	246.0 ± 54.9	0.7 (− 40.4, 41.8)	45.3 (− 0.6, 91.3)	30.3 (− 14.8, 75.4)	0.03	0.005	**0.03**
Carbohydrate, %	44.9 ± 8.0	45.3 ± 5.1	46.1 ± 8.6	48.9 ± 7.3	48.3 ± 6.0	48.2 ± 6.8	1.7 (− 4.2, 7.5)	2.4 (− 2.7, 7.6)	2.0 (− 4.7, 8.8)	0.24	0.51	0.89
Total sugars, g	101.1 ± 45.7	86.7 ± 36.6	87.9 ± 38.3	84.8 ± 33.5	96.0 ± 39.6	91.3 ± 25.9	− 8.6 (− 34.7, 17.6)	19.9 (− 8.7, 48.5)	14.9 (− 13.3, 43.1)	0.27	0.35	**0.006**
Fat, g	96.4 ± 37.3	77.6 ± 32.4	78.6 ± 33.2	78.8 ± 26.7	71.2 ± 28.4	76.4 ± 37.7	− 5.1 (− 18.3, 8.2)	7.2 (− 11.9, 26.2)	6.8 (− 21.0, 34.7)	0.58	0.0003	0.16
Fat, %	35.5 ± 6.0	34.4 ± 3.8	33.3 ± 5.9	33.6 ± 6.1	30.7 ± 5.1	31.7 ± 7.2	− 1.6 (− 6.2, 2.9)	−3.0 (− 8.0, 2.0)	−1.6 (− 6.5, 3.4)	0.14	0.02	0.64
Saturated Fat, g	31.5 ± 14.5	26.8 ± 12.3	24.7 ± 11.0^†^	24.4 ± 8.5	23.6 ± 10.6	25.4 ± 11.6	−3.8 (− 11.1, 3.5)	0.7 (− 7.3, 8.8)	3.8 (− 4.1, 11.8)	0.90	0.03	**0.03**
Protein, g	104.9 ± 37.5	94.1 ± 36.5	90.2 ± 32.1^†^	87.6 ± 23.1	94.1 ± 28.3	90.0 ± 26.1	−7.8 (− 26.3, 10.6)	9.8 (− 10.6, 30.3)	6.2 (− 13.8, 29.3)	0.61	0.07	**0.03**
Protein, %	17.5 ± 3.7	19.1 ± 4.1	17.4 ± 3.8	16.9 ± 3.4	18.7 ± 3.4	17.5 ± 3.4	− 0.9 (− 4.0, 2.1)	−0.9 (− 4.2, 2.4)	−0.5 (− 3.8, 2.8)	0.44	< 0.0001	0.87
Fiber, g	23.3 ± 9.3	21.0 ± 9.4	22.3 ± 9.3	24.0 ± 11.7	23.1 ± 12.2	23.8 ± 11.0	2.6 (− 5.8, 10.9)	5.1 (− 3.8, 13.9)	2.4 (− 6.3, 11.9)	0.22	0.03	0.45
Potassium, mg	3275.1 ± 1049.6	3068 ± 1241.8	2973.1 ± 900.2	3139.8 ± 1078.2	3328.1 ± 1295	3212.9 ± 1189.2	173.0 (− 458.1, 804.1)	673.4 (− 209.6, 1556.4)	500.2 (− 305.4, 1305.9)	0.047	0.14	0.19
Magnesium, mg	371.1 ± 139.9	327.9 ± 138.4	356.7 ± 138.5	363.7 ± 121.7	335 ± 149.5	347.3 ± 141.7	23.4 (− 70.9, 117.7)	54.2 (− 45.7, 154.1)	19.8 (− 79.0, 118.7)	0.29	0.007	0.36
Calcium, mg	998.9 ± 430.9	954.7 ± 452.0	793.5 ± 317.2	901.5 ± 254.9	1103.8 ± 468.9	961.1 ± 442.7	−5.7 (− 228.3, 216.8)	277.6 (− 80.7, 635.9)	282.5 (− 2.9, 567.1)	0.01	0.04	**0.02**
Vitamin C, mg	120.3 ± 47.4	129.2 ± 71.7	120.7 ± 77.6	139.6 ± 87.1	167.5 ± 81.3	138.2 ± 91.7	20.9 (− 39.0, 80.7)	37.3 (− 29.0, 103.5)	13.2 (− 52.3, 78.7)	0.17	0.34	0.60
NRF 9.3	46.1 ± 9.3	49.5 ± 9.6	47.7 ± 11.4	48.6 ± 8.0	50.0 ± 8.7	48.9 ± 8.4	0.7 (− 7.1, 8.6)	− 0.3 (− 8.6, 8.0)	−0.6 (− 8.8, 7.7)	0.98	0.15	0.79

*PRE* before the intervention (baseline), *POST 1* immediately after the intervention (week 9), *POST 2* 3–6 months after the intervention, *G x T* Group by time interaction, *NRF* Nutrient-Rich Food Index, *V/F* whole vegetables and fruits, *DP* Dairy products, *POST 1* four families did not complete their 3-day dietary record, *POST 2* three families did not complete their 3-day dietary record

^1^Values are presented as unadjusted means ± standard deviation

^2^Values are presented as adjusted means (95% confidence interval)

^†^Within-group significant differences vs. PRE

**Table 4:** BMI and BMI z-score at Baseline (PRE), Immediately After the Intervention (POST 1) and 3–6 Months After (POST 2) for Parents and their Children

	Groups^1^		Difference vs. control^2^	*P* value		
	Control	Intervention		Group	Time	Group x Time
**Parents, BMI (n, C/I)**				0.60	0.34	0.24
PRE (*n* = 75, 31/44)	28.62 ± 4.04	28.25 ± 4.49	−0.60 (− 3.42, 2.22)			
POST 1 (*n* = 66, 26/40)	28.25 ± 4.33	28.67 ± 4.70	−0.34 (− 3.17, 2.49)			
POST 2 (*n* = 55, 19/36)	28.39 ± 4.58	28.47 ± 4.89	−0.62 (− 3.45, 2.22)			
**Children, BMI z-score (n, C/I)**				0.57	0.05	0.29
PRE (*n* = 87, 39/48)	1.13 ± 0.84	0.95 ± 0.82	−0.11 (−0.64, 0.41)			
POST 1 (*n* = 77, 32/45)	0.91 ± 0.82	0.93 ± 0.85	−0.06 (− 0.59, 0.47)			
POST 2 (*n* = 65, 24/41)	0.87 ± 0.85	0.86 ± 0.94	−0.14 (− 0.67, 0.40)			

^1^Values C/I: Control, Intervention are presented as means ± standard deviation

^2^Values are presented as adjusted means (95% confidence interval)

## Discussion

Effective broad-reach interventions to promote healthy eating and prevent or reduce childhood obesity are highly needed. This study tested the effect of a family web-based intervention, which includes a nutrition challenge and involves the whole family, on vegetable and fruit (V/F) and dairy product (DP) consumption, nutrient intakes, diet quality and BMI or BMI z-scores. The results of this study showed that, compared to general nutritional guidelines, this family web-based nutrition intervention had a beneficial effect primarily on DP intakes in children and their parents. There was, however, no significant difference in changes in V/F, diet quality, BMI or BMI z-scores between the intervention and control groups.

Family Nutriathlon focuses on the consumption of V/F and DP yet, results indicated a beneficial effect of the intervention mainly on DP compared to general nutrition guidelines. A slight increase in fruit juice was also observed in parents in response to the intervention compared to control. Other parent-focused web-based interventions in similar age groups (9–16 y) reported a beneficial effect on certain dietary components such as V/F [38], while others did not [39, 40]. None have reported beneficial effects on DP intakes or overall diet quality. Moreover, only a few studies included the web as the sole intervention modality (similar to our study). One study examining the impact of a web-based intervention that included goal settings over 8 months in Chinese-American adolescents and their families found a significant increase in V/F intakes and physical activity practices and a decrease in the waist-to-hip ratio, but not BMI [38]. However, the control group did not include any intervention on nutrition or physical activity. The inclusion of a control group which received general nutrition guidelines from a registered dietitian may partly explain why there were few differences between our intervention and control groups. While this choice has some methodological advantages (e.g. helping to identify the most effective key features of an intervention, permitting blinding of the trials for the participants, ethical option for families struggling with obesity), it can also reduce the differences in outcomes between the intervention and control groups [41]. Accordingly, studies that included a control group with general nutrition guidelines and follow-ups with a professional (e.g. dietitian or physician) did not observe an improvement in V/F intakes in children compared to the control group [39, 40]. Another hypothesis to explain the modest improvement in V/F and nutrients is that baseline intakes in children and parents were already good. Thus, the initial eating habits of our population and the fact that the participants in the control group received a minimal intervention may explain the modest effect of Family Nutriathlon on outcomes.

Families increased their DP intake from 0.5–0.6 servings/day combined with a tendency to increase calcium intake in children (*P* = 0.058) compared to those exposed to general nutritional guidelines. This is concordant with a systematic review, demonstrating that interventions encouraging intakes of DP or calcium alone, without promoting other healthy eating habits, were more effective compared to those promoting dairy within the context of a healthy diet [42]. Yet, these interventions were not necessary for family web-based interventions. It is difficult to compare our results with other family web-based nutrition studies as DP are not frequently targeted and reported as an outcome. The effectiveness of Family Nutriathlon on DP consumption could also be related to specific goal settings towards DP in the intervention compared to the control group. Although the recommendation in the control group was to “drink low-fat milk or alternatives” every day, the recommendation was not specific to DP *per se,* but a more general strategy to include good sources of protein. Another hypothesis to explain the improvement in DP is that baseline intakes of DP in both children and parents were less optimal compared to baseline intakes of VF in both groups (i.e. about 1–2 servings/day for DP vs 5–6 servings/day for V/F). Thus, there was greater room for improvement in DP in both parents and children compared to the recommended servings per day in Family Nutriathlon (i.e. goal of 3 servings/day for DP). This may have been translated to increased motivation towards DP consumption within families. Moreover, because DP are easier to prepare than V/F, which may require cutting or cooking, it may have been easier for each family member, especially children, to increase their consumption of DP, suggesting increased autonomy of each individual towards DP consumption. Nevertheless, the consumption of DP decreased at follow-up for children and parents in Family Nutriathlon. The reason for this is not known, but it is possible that not being part of a challenge anymore decreased children’s motivation. Thus, as included in Family Nutriathlon’s, specific family “goal setting” through a family challenge appears to be a good approach to develop nutritional strategies in children and adults which permit an increase in dairy intakes, at least in the short term.

Even though the intervention favors the consumption of nutrient-dense foods such as DP, it had a modest impact on nutrient intakes and no effect on global diet quality was observed. Overall diet quality is not often assessed in family web-based interventions [10] which makes it difficult to compare these results with other studies. A recent review focussing on effectiveness of nutritional strategies on improving diet quality in children (which only include three studies with parents out of twelve), found that most studies assessed diet quality or dietary patterns mainly by focussing on an increase consumption of foods considered as healthy (fruits and vegetables) and a decrease in those less healthy (sugary drinks, less healthy snacks) [43]. In our study, overall diet quality was assessed using the NRF9.3, which has been validated with the Healthy Eating Index [32, 33]. However, NRF9.3 may not be the optimal diet quality index for DP, since they contain a high amount (or quantity) of nutrients to encourage but also a high amount of nutrients to limits such as saturated fat. In future family web-based nutrition intervention studies, the possibility of incorporating diet quality indices based on food groups and/or nutrients into the evaluation methods should be considered more to evaluate the global effect of a nutrition intervention.

There was no impact of the Family Nutriathlon on children’s BMI z-scores or parents’ BMI. While parental BMI did not change, children’s BMI z-scores decreased, but similarly to children following the general nutrition guidelines. This effect in children was also not dependant on obesity status. Even though most of the families had at least one child with overweight or obesity, the objective was not to lose weight *per se* but to improve eating habits in the long term and prevent obesity. These results are in line with previous studies on parent-focused eHealth interventions which reported no effect of the interventions on children’s BMI or z-BMI between intervention and control groups despite some beneficial effects on physical activity and nutrition behaviours [10]. It is also interesting to note that the increase in DP consumption was not associated with a concomitant change in body weight. It is possible that even if participants consumed more DP, energy density was most likely decreased as previously shown [19]. A study showed that DP drinks consumed before and with a meal have more favorable effects on appetite, satiety hormones, and short-term food intake than other sugar-sweetened beverages [44]. This is also concordant with observational studies showing that childhood dairy consumption is associated with a decreased risk of overweight/obesity [21, 22]. Dairy components such as protein (i.e. whey protein) [45] and calcium [46], shown to improve appetite control, may lead to the obesity preventive effects of dairy. Because body weight change is a long-term process and depends on multiple factors, the intervention period may not have been long enough to observe differences in body weight changes between groups. Taken together, these results suggest that the increase in DP consumption observed in children and parents in response to Family Nutriathlon, if maintained, could help to manage body weight in children over the long term yet, further studies need to confirm this hypothesis.

The present study has both strengths and limitations. The evaluation of the impact of an innovative family nutrition program using the internet as a main mode of intervention on children and their parents represents a strength. Several studies in this field have used web-based modalities combined with other modes of intervention (e.g. face-to-face, workbooks) [10] and to our knowledge, none assessed outcomes in children between 8 and 16 years nor their parents. This study contributes to the field of family web-based interventions in childhood obesity prevention and management by identifying one specific family web-based intervention, i.e. a nutrition challenge for the whole family which can stimulate the consumption of dairy through goal-setting, self-monitoring, feedback, identification of barriers, solutions and social support. The mode of delivery of intervention (web-based intervention with a platform) is also cost-effective and can help overcome barriers such as availability, cost, transportation, time constraints of many families [47] as well as sanitary measures related to the context of the pandemic. Considering that the intervention was implemented among families under real-life conditions, we believe that this intervention could easily be expanded to reach a greater proportion of the population while retaining effectiveness. Moreover, this intervention could represent a useful approach for registered dietitians who work with families and children at risk of obesity. Most importantly, this intervention which targets the entire family and not only the child exhibiting overweight or obesity, can help to decrease stigmatization around obesity and favor more positive eating behaviors of the children and their parents in the long-term. Nevertheless, some limitations of this type of intervention include access to computers and the internet which may be a problem in low-income families [48] and technological issues such as login problems, incompatibility of the platform with some devices such as tablets or phones [27]. Elevated attrition rate of web-based interventions may also be a factor [49] which could explain, in part, our results. Interestingly, families in the Family Nutriathlon had a higher attendance rate (adherence) to regulation periods/sessions and a lower drop-out rate compared to families in the control group (attendance at all regulation periods/sessions = 82% vs 53% and drop-out rate = 17% vs 32%, respectively). In addition, the drop-out rate found in our study was similar to other family web-based interventions (ranged between 12 to 29%) [10]. Another limitation of our study is the inability to identify specific food groups (e.g. type of dairy products) that was increased in families. Studies have shown that types of dairy (e.g. yogurt vs. cheese vs. milk, regular vs. low-fat) may have a different impact on body weight [50] and cardiometabolic health [51, 52]. Like many studies in this field, V/F and DP consumption was self-reported and were exposed to social desirability bias; a bias generally occurring in nutrition studies because those interested in nutrition generally have a better diet quality and higher socioeconomic levels. This was more specifically observed for mean baseline intake of VF in children and their parents which seemed to be slightly higher compared to the general population (5.2 and 6.0 serving/d in the present study compared to 4.2 and 4.6 serving per/day in the children and adults from the Canadian population) [53]. Yet, this bias was the same in both groups since both groups received an intervention by a registered dietitian and were blinded to the intervention. It is also important to note that Family Nutriathlon was not specifically based on a behavioural change theory, but nonetheless included many key components in line with the self-determination theory and behavioural change techniques. Lastly, considering the small sample size and the short-term intervention, we acknowledge that the need to evaluate Family Nutriathlon in a larger sample and over a longer period with additional regulation periods/sessions with a dietitian (e.g. twice a year) on behavioral change over time.

In conclusion, this family web-based nutrition intervention had a primarily modest effect on dairy product intakes in children and their parents, at least in the short term. Moreover, compared to a general nutrition intervention, this nutrition challenge which included the whole family appears to favor adherence to a nutrition intervention. Considering the challenge to access to obesity prevention and treatment programs, this intervention represents one flexible and cost-effective tool for health professionals which has the potential to involve a larger number of families compared to traditional interventions. Longer term studies which include this web-based family challenge as part of a multi-component intervention is needed to assess the sustainability of these changes in the prevention and treatment of obesity

## Supplementary Information

**Additional file 1.**

## Data Availability

The datasets analysed during the current study are available from the corresponding author on reasonable request and pending approval from the authors as well as the funding agency.

## Abbreviations

BMI: Body Mass Index
DP: Dairy Products
V/F: Vegetable and Fruit
